# Neuroacanthocytosis Syndromes in an Italian Cohort: Clinical Spectrum, High Genetic Variability and Muscle Involvement

**DOI:** 10.3390/genes12030344

**Published:** 2021-02-26

**Authors:** Alessandro Vaisfeld, Giorgia Bruno, Martina Petracca, Anna Rita Bentivoglio, Serenella Servidei, Maria Gabriella Vita, Francesco Bove, Giulia Straccia, Clemente Dato, Giuseppe Di Iorio, Simone Sampaolo, Silvio Peluso, Anna De Rosa, Giuseppe De Michele, Melissa Barghigiani, Daniele Galatolo, Alessandra Tessa, Filippo Santorelli, Pietro Chiurazzi, Mariarosa Anna Beatrice Melone

**Affiliations:** 1 Istituto di Medicina Genomica, Università Cattolica del Sacro Cuore, 00168 Roma, Italy; alessandro.vaisfeld@hotmail.com; 2 Department of Advanced Medical and Surgical Sciences, 2nd Division of Neurology, Center for Rare Diseases and Interuniversity Center for Research in Neurosciences, University of Campania “Luigi Vanvitelli”, 80131 Naples, Italy; giorgiabruno990@gmail.com (G.B.); stracciagiulia@gmail.com (G.S.); clemente.dato@gmail.com (C.D.); giuseppe.diiorio@unicampania.it (G.D.I.); simone.sampaolo@unicampania.it (S.S.); marina.melone@unicampania.it (M.A.B.M.); 3 Fondazione Policlinico Universitario “A. Gemelli” IRCCS, UOC di Neurologia, 00168 Roma, Italy; martina.petracca@gmail.com (M.P.); annarita.bentivoglio@policlinicogemelli.it (A.R.B.); mariagabriella.vita@policlinicogemelli.it (M.G.V.); francescobove86@gmail.com (F.B.); 4 Dipartimento Universitario di Neuroscienze, Università Cattolica del Sacro Cuore, 00168 Rome, Italy; serenella.servidei@unicatt.it; 5 Fondazione Policlinico Universitario “A. Gemelli” IRCCS, UOC di Neurofisiopatologia, 00168 Rome, Italy; 6 Department of Neurosciences and Reproductive and Odontostomatological Sciences, Federico II University, 80138 Naples, Italy; dr.silviopeluso@gmail.com (S.P.); anna_derosa@libero.it (A.D.R.); demichel@unina.it (G.D.M.); 7 Molecular Medicine, IRCCS Fondazione Stella Maris, 56128 Pisa, Italy; mely.b91@hotmail.com (M.B.); daniele.galatolo1408@gmail.com (D.G.); atessa@fsm.unipi.it (A.T.); filippo3364@gmail.com (F.S.); 8 Fondazione Policlinico Universitario “A. Gemelli” IRCCS, UOC Genetica Medica, 00168 Roma, Italy; 9 Sbarro Institute for Cancer Research and Molecular Medicine, Center for Biotechnology, Temple University, Philadelphia, PA 19122-6078, USA

**Keywords:** neuroacanthocytosis syndromes, chorea-acanthocytosis, McLeod syndrome, *VPS13A* gene, *XK* gene

## Abstract

Neuroacanthocytosis (NA) syndromes are a group of genetically defined diseases characterized by the association of red blood cell acanthocytosis, progressive degeneration of the basal ganglia and neuromuscular features with characteristic persistent hyperCKemia. The main NA syndromes include autosomal recessive chorea-acanthocytosis (ChAc) and X-linked McLeod syndrome (MLS). A series of Italian patients selected through a multicenter study for these specific neurological phenotypes underwent DNA sequencing of the *VPS13A* and *XK* genes to search for causative mutations. Where it has been possible, muscle biopsies were obtained and thoroughly investigated with histochemical assays. A total of nine patients from five different families were diagnosed with ChAC and had mostly biallelic changes in the *VPS13A* gene (three nonsense, two frameshift, three splicing), while three patients from a single X-linked family were diagnosed with McLeod syndrome and had a deletion in the *XK* gene. Despite a very low incidence (only one thousand cases of ChAc and a few hundred MLS cases reported worldwide), none of the 8 *VPS13A* variants identified in our patients is shared by two families, suggesting the high genetic variability of ChAc in the Italian population. In our series, in line with epidemiological data, McLeod syndrome occurs less frequently than ChAc, although it can be easily suspected because of its X-linked mode of inheritance. Finally, histochemical studies strongly suggest that muscle pathology is not simply secondary to the axonal neuropathy, frequently seen in these patients, but primary myopathic alterations can be detected in both NA syndromes.

## 1. Introduction

Chorea-acanthocytosis (ChAc, OMIM #200150) is a rare hereditary neurodegenerative condition, characterized by the young adult-onset of involuntary choreiform movements and red blood cells (RBCs) acanthocytosis. The phenotypic spectrum is relatively wide and variable, encompassing psychiatric symptoms, cognitive impairment, peripheral neuropathy, myopathy and epilepsy [1]. Besides chorea, other motor manifestations may include facial and oromandibular dystonia, tics, parkinsonism and postural tremor. Although the misshaped RBCs are considered the diagnostic hallmark, the acanthocytes can sometimes be absent or even make a late appearance in the course of the disease. Other common laboratory findings in ChAc include increased levels of creatine kinase and liver enzymes. Together with the X-linked McLeod syndrome (MLS, OMIM #300842), due to loss-of-function (LOF) variants of the *XK* gene (*314850), ChAc accounts for the “core” neuroacanthocytosis (NA) syndromes, defined by the combination of RBC acanthocytosis and basal ganglia-related neurological disorders [1,2]. ChAc represents one of the most common differential diagnoses of Huntington’s disease (HD). The inheritance pattern of ChAc is autosomal recessive and patients are either homozygous or compound heterozygous for LOF variants in the *VPS13A* gene (OMIM *605978) encoding chorein, a large protein involved in the intracellular vesicle trafficking [1]. Mammalian *VPS13A* belongs to a small gene family that codes for ubiquitously expressed proteins derived by the duplication of an ancestral yeast gene [3]; pathogenic variants in the other genes of this family underlie other neurologic conditions such as Cohen syndrome (*VPS13B*), early-onset autosomal recessive Parkinson’s disease (*VPS13C*) and spinocerebellar ataxia with saccadic intrusions (*VPS13D*). Although ChAc inheritance is generally accepted as autosomal recessive, patients with possible autosomal dominant ChAc have occasionally been reported [4,5,6,7] but remain questionable in the pre-exome sequencing era. Indeed, a second *VPS13A* variant may initially go unnoticed, as in the affected siblings reported by Saiki et al. [4] where a second *VPS13A* variant was subsequently identified [8].

We describe here a series of Italian patients with NA syndromes recruited as part of a multicenter study, that were screened for pathogenic variants in the *VPS13A* and *XK* genes. In a total of six families, we identified eight different *VPS13A* variants, mostly unreported, and a single exon deletion of the *XK* gene. The clinical description, including detailed muscular pathology, laboratory findings and imaging features are reported. 

## 2. Materials and Methods

### 2.1. Patient Series

The patients with NA syndromes described here come from clinical–diagnostic evaluations carried out in the neurology departments of three different University hospitals in Italy. All examiners used commonly adopted scales according to shared criteria to assess the core phenotype and disability score i.e. the Unified Huntington’s Disease Rating Scale (UHDRS), the Montreal Cognitive Assessment (MoCA), the Mini Mental State Evaluation (MMSE), and the Manual Muscle Test (MMT)-Medical Research Council (MRC) grading scale. With the exception of 2 patients, all other cases described here were not previously reported. This study was conducted according to the Declaration of Helsinki and written informed consent was obtained from all participants.

### 2.2. Molecular Analysis 

The molecular diagnostics of probands, and other affected or unaffected family members, were performed between 2016 and 2018 in the same laboratory. Total DNA was isolated from peripheral blood using a MagPurix automatic extractor. All exons and flanking regions of the *VPS13A* and *XK* genes were amplified from genomic DNA and customized primers (listed in the Supplementary Material). Amplicons were purified using Exo-Sap, labeled using a BigDye Terminator v3.1 Cycle sequencing kit (Applied Biosystem, Foster City, CA, USA) and directly sequenced on an ABI 3500 Genetic Analyzer (Applied Biosystem, Foster City, CA, USA). Total RNA was purified from blood and was reversely transcribed using the First Strand cDNA Synthesis Kit (Roche, Hamburg, Germany) according to the manufacturer’s random primer protocol. The effect of the *VPS13A* intronic variant on splicing in family B was examined by RT-PCR using gene specific primers and Sanger sequencing. Primer sequences have been reported in the Supplementary Material and PCR conditions are available upon request. The identification of exon 1 deletion in the *XK* gene in family F was obtained by quantitative real-time PCR (qPCR) in a MicroAmp^®^ optical 96-well plate using exon specific oligonucleotide primers and SYBR Green mastermix (ThermoFisher, Waltham, MA, USA). The relative quantification of the copy number was performed according to the comparative method (2—ΔΔCt) [9]. The mean result of three independent experiments run in triplicate was compared with that of normal controls whose expression was arbitrarily attributed the value of 1. Western blotting was performed in some patients on blood in order to detect the VPS13A protein using the anti-chor1 antibody as described previously [10].

### 2.3. Muscle Studies

#### 2.3.1. Chorea-Acanthocytosis

Open muscle biopsies on patients from two different families with ChAC were performed at the Department of Neurology of the University of Campania “Luigi Vanvitelli”: patient II:4 from family A had a biopsy on the vastus lateralis of the right quadriceps, while patients II:2 and II:3 from family D were biopsied on the left deltoid. Muscle tissues were snap-frozen in isopentane, pre-cooled in liquid nitrogen and stored at −260 °C until sectioning. 7 and 10-µm-thick serial cryo-sections were used for histology and histochemistry and stainings included: hematoxylin eosin (H&E), Gomori trichrome, periodic acid Schiff (PAS), Sudan III, adenosine triphosphatase (ATPase) at pH 9.6, 4.6 and 4.3, AP (acid phosphatase) and oxidative enzymes such as nicotinamide adenine dinucleotide-tetrazolium reductase (NADH-TR), succinate dehydrogenase (SDH) and cytochrome oxidase (COX). Qualitative and quantitative light microscopy studies were carried out on muscle slices using a Nikon Eclipse Ni^®^ photomicroscope equipped with the NIS Elements F4.30.00 image analyses system. 

#### 2.3.2. McLeod Syndrome

Following the same protocol described above, muscle studies were also performed on three individuals affected by McLeod syndrome: F-III:4 had biopsy on the right peroneus brevis at the Neurology Division of the “Federico II” University of Naples, while patients F-III:5 and F-III:9 were biopsied on the right quadriceps, respectively, at the Department of Neurology of University of Campania “Luigi Vanvitelli” and at the Neurology Unit of Padua University.

## 3. Results

Table 1 summarizes the clinical data of all 12 patients belonging to the six families (A–F). Individual patients’ histories are described in detail in the Supplementary Material, while muscle studies performed on patients A-II:4, D-II:2, D-II:3, F-III:4, F-III:5 and F-III:9 are reported in a dedicated section.

Table 2 lists the *VPS13A* variants identified in the first five families (A–E) next to their frequency in the Genome Aggregation Database (gnomAD) and the expected protein changes.

### 3.1. Family A (Chorea-Acanthocytosis)

**Figure 1:**
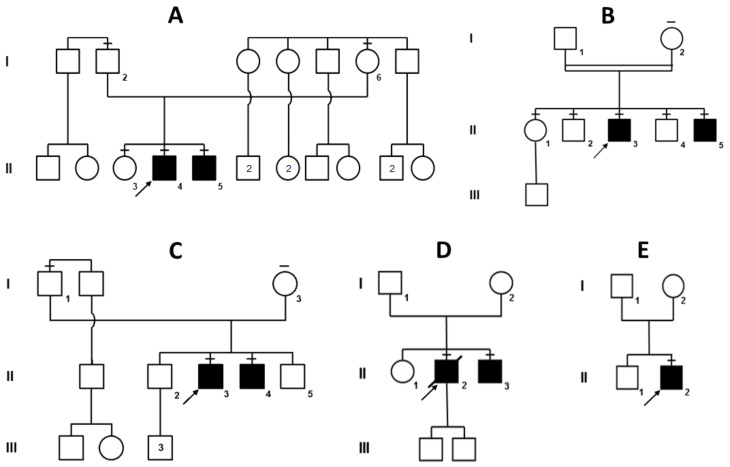
Pedigrees of the five choreo-acanthocytosis families (**A–E**), all with a clear recessive mode of inheritance. However, consanguineity was confirmed only for pedigree B (parents are second-degree cousins) and suspected for pedigree D (both parents carry the same *VPS13A* variant). Affected individuals are shaded in black. Family members that were sequenced are indicated by a dash on top of their symbol.

This pedigree (Figure 1A), originating from two Vesuvian territories, includes two affected individuals. Molecular analysis of the *VPS13A* gene in the proband A-II:4 confirmed the ChAc diagnosis and revealed the presence of three variants: c.3817C>T, c.1114_1115delAA and c.3592A>C; the last two were found to be in cis on the maternal copy of the gene (Supplementary Figure S1). The first variant (inherited from the father) introduces a stop codon in position 1273 (p.R1273*) and the second (inherited from the mother) is a frameshift variant disrupting the protein after residue 372 (p.K372Vfs*4). While these two variants are both pathogenic and clearly account for the patients’ phenotype, a third variant (c.3592A>C) has been co-inherited with the maternal frameshift, causing a likely benign missense change (p.K1198Q). The same variants have been detected in the younger brother (A-II:5) while their older sister (A-II:3) carries only the paternal c.3817C>T (p.R1273*) nonsense variant. 

### 3.2. Family B (Chorea-Acanthocytosis) 

This consanguineous pedigree (Figure 1B) originates from a town near Brindisi, in the south-eastern tip of Italy, and the parents are second cousins. Molecular analysis eventually revealed a homozygous *VPS13A* variant in both affected brothers B-II:3 and B-II:5 (c.3339+4_3339+10delinsTATAGCTGTTATATAAAATTATTTAA). As shown in Supplementary Figure S2, this insertion–deletion, close to the splice donor site in intron 31, alters the canonical splicing of *VPS13A* mRNA, likely resulting in protein loss-of-function. The two other brothers (II:2 and II:4) are both heterozygous carriers, like their mother I:2. The older sister II:1 turned out to be homozygous for the wild-type allele, while the father I:1 (an obligate carrier) declined genetic testing. 

### 3.3. Family C (Chorea-Acanthocytosis)

This non-consanguineous family has two probands, C-II:3 and C-II:4 (Figure 1C). Only in 2017, at the age of 41, molecular analysis of *VPS13A* was eventually performed on C-II:4, revealing a compound heterozygosity for two nonsense variants c.1078C>T (p.Q360*) and c.7867C>T (p.R2623*). Sequences of the two variants are shown in Supplementary Figure S3. Shortly after his younger brother, C-II:3 also received molecular confirmation of his ChAc diagnosis and was found to carry the same two nonsense variants. Two other unaffected brothers (II:2 and II:5) have not been tested yet. 

### 3.4. Family D (Chorea-Acanthocytosis)

This family has two affected brothers (II:2 and II:3) and originates from Herculaneum, south-east of Naples, close to Mount Vesuvius (Figure 1D). Only the two affected brothers have been tested and the proband (D-II:2) has recently passed away. Although no parental consanguinity had been reported, *VPS13A* sequencing revealed a homozygous splicing variant (c.2512+2T>G) that abolishes the donor site of intron 24 Supplementary Figure S4). Genetic testing of the younger brother D-II:3 confirmed the presence of the same homozygous *VPS13A* variant. 

### 3.5. Family E (Chorea-Acanthocytosis)

The proband (E-II:2) is the second son of a non-consanguineous marriage (Figure 1E). This patient, along with D-II:3, was previously reported by Peluso et al. [11], who made a biochemical diagnosis of ChAc since Western blotting had proven the absence of chorein, the VPS13A protein. Eventually *VPS13A* sequencing revealed that patient E-II:2 is a compound heterozygote for two different variants, namely, a 4-bp deletion (c.7736_7739del) causing a frameshift in exon 55 and an intronic substitution close to the acceptor site of intron 26 (c.2825-10T>G). Disruption of normal mRNA splicing has been confirmed with cDNA sequencing of the relevant *VPS13A* exons (Supplementary Figure S5). 

### 3.6. Family F (McLeod Syndrome)

**Figure 2:**
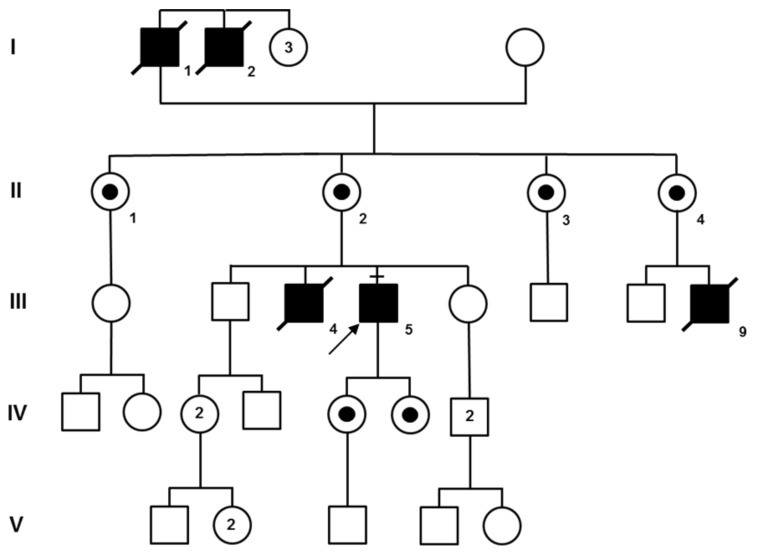
Pedigree of family F is compatible with X-linked inheritance. The molecular test of III:5 indirectly confirmed the diagnosis of McLeod syndrome for the other patients (I:1, I:2, III:4, and III:9) who had already died. Obligate carriers are indicated with a small black circle within their symbol.

This large pedigree (Figure 2) originates from the metropolitan area of Venice and all patients but the proband F-III:5 are now dead. The familial history clearly suggested X-linked inheritance and, in fact, the absence of the Kell antigen on red blood cells confirmed this suspicion. Genetic testing eventually proved that proband III:5 was positive for a hemizygous deletion of exon 1 of the *XK* gene. As shown in the pedigree, four other individuals have been reported to be affected by family members and died before DNA analysis was available. However, III:4 and III:9 were subjected to muscle biopsy as described in the following section.

### 3.7. Muscle Studies

**Figure 3:**
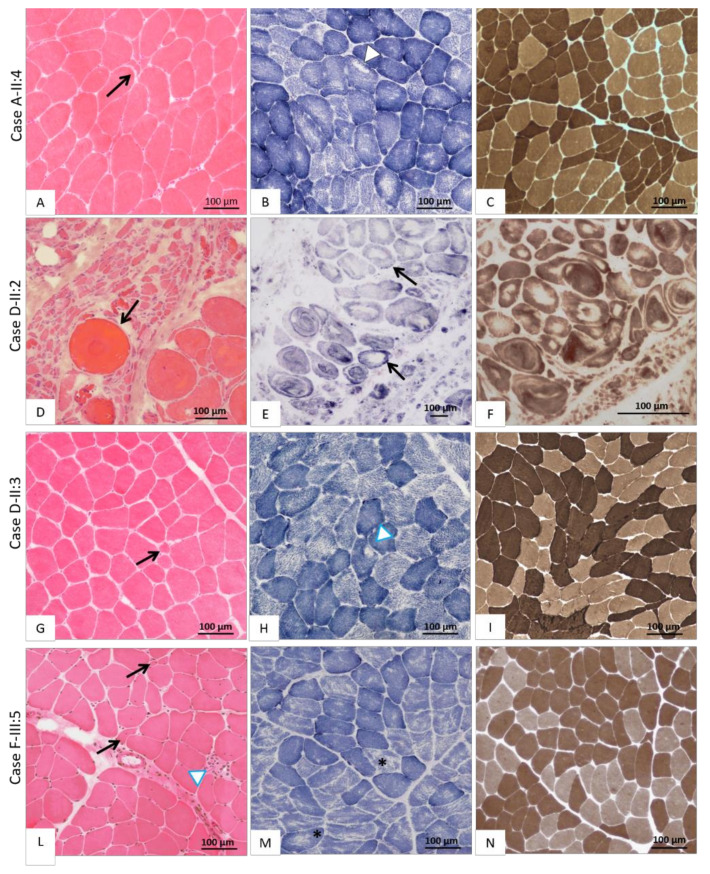
Histological findings on muscle biopsy. The first column shows images of muscle stained with haematoxylin-eosin (H&E), the second with succinate dehydrogenase (SDH) and the third with myosinATPase at pH 9.4. (**A**–**C**) Case A-II:4 affected by ChAc: (**A**) moderate fiber size variability with some hypo-atrophic angulated fibers (black arrow) next to large hypertrophic fibers; (**B**) the great majority of type 1 fibers display multi-minicores and some poorly structured cores (white head-arrow); (**C**) atrophic fibers are organized in small-medium groups (type-grouping phenomena). (**D**–**F**) Case D-II:2 affected by ChAc; (**D**) very severe fiber size variability with numerous atrophic angulated fibers and atrophic fascicles, next to giant hypertrophic round fibers (black arrow); (**E**) severe myofibrillar architecture disruption with bizarre fiber shape, vortex fibers and presence of unstructured cores in most fibers (black arrow); (**F**) disruption of muscle architecture where fiber types are hardly distinguishable. **(G–I)** Case D-II:3 affected by ChAc; (**G**) poor fiber size variability with some hypotrophic fibers (black arrow); (**H**) rare structured core fibers (white arrowhead); (**I**) fibers grouping according to their histochemical type thus suggesting central denervation and reinnervation. (**L**–**N**) Case F-III:5 affected by McLeod syndrome; (**L**) fiber size variability with numerous clumps of naked nuclei (black arrow) and an infiltrate of inflammatory cells (white head-arrow); (**M**) numerous moth-eaten fibers (black stars); (**N**) some type grouping phenomena.

Muscle biopsies were performed in three patients with ChAc and in three individuals of the MLS family; the main histological findings are illustrated in Figure 3. 

#### 3.7.1. Chorea-Acanthocytosis

In patient A-II:4 (Figure 3A–C) the muscle biopsy disclosed predominantly neurogenic alterations, with fiber caliber variability due to small groups of atrophic angulated fibers, next to some hypertrophic type I and type II fibers at myosinATPase pH 9,4; rare unstructured core fibers were also detected, suggesting chronic denervation. Muscle specimens obtained from the two affected siblings of family D showed a significantly different degree of muscle neurogenic degeneration. In patient D-II:2 (Figure 3D–F) a severe variability of fiber size was observed due to large groups of atrophic fibers (Ø < 20 µm) intermingled with several giant fibers (Ø > 120 µm). Splitting phenomena and the severe disruption of myofibrillar architecture in the form of whorled and bizarre-shaped fibers were also seen with oxidative enzymes staining (Figure 3E–F). The biopsy of Patient D-II:3 (Figure 3G–I) showed a histological picture similar to A-II:4, characterized by a slight variability in fiber size, atrophy of both type I and II fibers, and fiber-type grouping phenomena. 

#### 3.7.2. McLeod Syndrome

The two brothers (F-III:4 and F-III:5) displayed diffuse neuropathic muscle changes with a modest increase in endomysial connective tissue. Moreover, in case F-III:5 (Figure 3L–N), diffuse inflammatory infiltrates were evident along with some moth-eaten and core-like fibers, while in patient F-III:4, a single fiber with a “rimmed vacuole” was detected (pictures not available). Finally, in the biopsy of patient F-III:9 (pictures not available), myopathic changes with a moderate variability in muscle fiber size were detected, consisting in prevalence of type I and IIC fibers and increased central nuclei.

## 4. Discussion

Here, we report 12 Italian patients with a molecular diagnosis of NA, nine belonging to five ChAC families (carrying bi-allelic variants of the *VPS13A* gene) and three belonging to the same family with McLeod syndrome (harboring a deletion of exon 1 of the X-linked *XK* gene). The clinical history of these patients, summarized in Table 1 and detailed in Supplementary Material (Table S1 and Table S2), allowed us to highlight some aspects of the NA syndromes. Due to the small number of patients with MLS (Family F), a clinical comparison with ChAc patients (Families A–E) does not add much to what has already been published [15,16]. As already reported, also in our series the ChAc phenotype is variable among patients, even within the same family: due to the high variability and low prevalence, NA is underreported and diagnosed with a clinically significant delay [17,18]. Among the core symptoms, tics and choreic movements, with a prevalence of orofacial dyskinesia, stand out. This is a first major phenotypic difference with the most frequent form of hereditary chorea, i.e., Huntington’s disease (HD), in which choreic movements mainly affect limbs at onset; another distinguishing feature is the occurrence of epilepsy in ChAc (even at an early stage), while epilepsy is rare and late in typical adult-onset HD patients. On the other hand, psychiatric symptoms are as frequent as those found in other hereditary choreas.

Muscle pathology of the NA syndromes is poorly understood, however, a distinction between ChAc and MLS must be made as they are two separate conditions. In fact, a neurogenic pattern at muscle biopsy was reported in most ChAc patients, though minor myopathic changes were observed in few cases [19,20]. Conversely, a primary myopathic pattern was reported in the MLS series [20]. Actually, the pathogenesis of muscle alterations in ChAc is still a matter of debate. Indeed, bizarre fibers, such as nucleus fibers, can be found in both dystrophic process and chronic denervation [21]. Whereas some studies focused on neurogenic muscle wasting in NA, little is known about the mechanisms underlying primary myopathy. Conformational and functional anomalies of tTGase products were proposed to alter the deformability of both erythrocytes and muscle membrane in ChAc [22,23]. Saiki et al. [24] proved the loss of chorein expression in the skeletal muscle of ChAc patients and suggested that this protein plays a role in mitochondrial activity. Since *VPS13A* is ubiquitously expressed, mutated chorein could be detected in all tissues of ChAc patients, including the skeletal muscle [25]. Undoubtedly, the pattern of fascicular atrophy found in patient D-II:2 (Figure 3D–F) allowed us to hypothesize that the second motor neuron body or the motor nerve roots may be the primary site of pathogenic events in ChAc, at least in some patients. However, the detection of whorled and bizarre shaped fibers (Figure 3E–F) suggests a concomitant primary myopathic process: as far as we know, these peculiar alterations have not been previously reported in ChAc patients. Furthermore, we note that muscle changes were very severe in the biopsy of patient D-II:2 (Figure 3D–F) compared to his brother D-II:3 (Figure 3G–I); although the two brothers share the same *VPS13A* splicing variant, this phenotypic heterogeneity cannot be explained only by disease duration, but genetic and/or epigenetic factors might be involved. The mixed neurogenic and myopathic pattern of muscle pathology in ChAc, already described in the literature, is further confirmed by our findings in A-II:2 (Figure 3A–C).

Let us now consider the other NA form, i.e., MLS, which was initially defined as a benign X-linked myopathy with acanthocytes [26], although Hewer et al. [27] claimed that it is neither benign nor a pure myopathy. Since a dystrophic pattern of myopathy was reported in some rare cases [28], an involvement of dystrophin (whose gene is 4 Mb upstream of *XK* on the X chromosome) was sought but not confirmed [29,30]. Immunocytochemical studies suggested that lack of XK expression in MLS may destabilize the normal muscle structure and function, but the protein role remains unclear [31]. The 444-amino acid long XK protein has 10 transmembrane domains, it belongs to the 4.1R multiprotein complex of the red blood cell membranes and is joined to the Kell protein by a disulphide bond [32]. The deletion of exon 1 of the *XK* gene identified in our family F (Figure 2), which had been reported at least once before [33], is expected to completely abolish the presence of the protein in all cells of hemizygous males. Finally, the clinical and muscular phenotype (Figure 3L–N) of family F confirms that MLS is a mixed neuropathic and myopathic condition, with variable severity of CNS involvement. Based on our findings and those of the literature, histological signs of primary myopathy along with less severe neurogenic changes should be considered more suggestive of MLS rather than of ChAc. 

We should now focus on the high genetic variability of *VPS13A* variants identified in our ChAc families (Figure 1), all of Italian origin: it is worth noting that, differently to what has been reported in the Japanese population [8,34], our patients do not present with recurrent variants (Table 2). Furthermore, the two most frequent Japanese variants (p.R1471* and p.V2738Afs*5), present in more than half of the patients [8,34], are absent in our series. In agreement with previous reports [34], where missense pathogenic variants are exceedingly rare, we also found three nonsense, two frameshift and three splice-site variants in our nine ChAc patients (Table 2). Only one missense variant (p.K1198Q) has been detected in Family A, but it is predicted to be likely benign and, most importantly, it was found in cis with the pathogenic frameshift variant (p.K372Vfs*4). 

As shown in Table 2, only two of the listed *VPS13A* variants were previously reported in ChAc patients; the first such variant (c.7867C>T) introduces a stop codon truncating chorein at arginine 2623 (p.R2623*). This variant was first identified by Dobson-Stone et al. [12] in the original Kentucky family, clinically described by Critchley et al. [35] in 1968 and was afterwards reported in several independent patients [8,13,14]. The other previously reported variant is a 2-bp deletion (c.1114_1115del) causing an early frameshift (p.K372Vfs*4), reported just once in the extensive series of Tomiyasu et al. [8]; interestingly, although they analyzed patients from 11 countries, the only patient carrying this variant (in homozygosity) was Italian. The c.1114_1115del frameshift might be therefore an Italian variant, also found in our family A from Naples where it associates in compound heterozygosity with the c.3817C>T nonsense variant (Supplementary Figure S1). The other six variants found in our nine ChAc patients are apparently novel, and three of them are not even found in the Genome Aggregation Database (gnomad.broadinstitute.org/, last accessed on 30 December 2020) [36]; this unexpected high genetic variability suggests that many undetected *VPS13A* variants are actually present in the Italian and possibly other European populations. Although Italy has a very diverse genetic structure due to its central position in the Mediterranean and the numerous migratory waves that has mixed its population over the millennia, we conclude from our study that *VPS13A* variant screening should be considered in patients with chorea (after HD has been excluded) even in the absence of acanthocyte studies.

## 5. Conclusions

To summarize, we reported a relatively large series of NA patients proving that ChAc and MLS, although rare, should be always considered in the differential diagnosis of suspected hereditary chorea. Depending on the laboratory techniques available, chorein dosage with Western blotting and Kell blood group expression may be tested first or DNA sequencing of the *VPS13A* and *XK* genes will be performed. Moreover, since a primary myopathy is now suspected not only in MLS but also in ChAc patients, it would be useful to perform a muscle biopsy and protein expression studies in all confirmed NA patients in order to thoroughly study skeletal muscle involvement in these conditions.

## Figures and Tables

**Table 1:** Main clinical, laboratory and histological features. Acanthocytes: percentage of acanthocytes on peripheral blood smear; Serum CK level: normal range 60–190 U/L; OCD: obsessive-compulsive disorder; CN: caudate nuclei; LL: lower limbs; MUP: motor unit potential; ChAc: chorea-acanthocytosis; MLS: X-linked McLeod syndrome; n.a: not available. * previously reported by Peluso et al. [11].

	A-II:4 (ChAc)	A-II:5 (ChAc)	B-II:3 (ChAc)	B-II:5 (ChAc)	C-II:3 (ChAc)	C-II:4 (ChAc)	D-II:2 (ChAc)	D-II:3 * (ChAc)	E-II:2 * (ChAc)	F-III:4 (MLS)	F-III:5 (MLS)	F-III:9 (MLS)
**Age at onset **	6	24	27	25	11	20	40	30	28	45	39	20
**Age at last update **	39	25	36	30	48	41	52 (dead)	46	53	59 (dead)	63	45 (dead)
**Onset ** **Symptoms**	Behavioral disorder	Anxiety disorder	Orofacial tics	Behavioral Disorder	Tics, seizures	Psychosis, OCD	Feeding dystonia, muscle weakness	Seizures	Mood and behavioral disorders	Muscle weakness	Seizures	Behavioral disorder
**Acanthocytes (%)**	7.3–19.2	8.3	8.6	3.1	16.4	28	17	13	40	9–10	18	5–7
**Serum CK level (U/L)**	600	1971	489–948	5564	872–3435	1180	3000	1522	1500	2500	1000	1000–8000
**Choreic movements ** **(onset age)**	YES (15)	No	YES (29)	No	YES (30)	YES (30)	YES (42)	No	No	YES (50)	YES (59)	YES (45)
**other ** **movement disorders**	Feeding dystonia	Akathisia, Tics	Tics	Tics	Facial dyskinesia, dystonia	Dystonia, bradykinesia, postural tremor	Feeding dystonia	No	Bradykinesia, tremor, motor slowing, dystonia	No	Buccal stereotypies	Oro-facial dystonia
**Seizures (onset age)**	YES (23)	No	No	YES (25)	YES (11)	No	No	YES (30)	No	No	YES (38)	YES (40)
**Psychiatric symptoms (onset age)**	YES (6)	YES (23)	No	YES (26)	No	YES (20)	No	No	YES (28)	No	No	YES (20)
**Brain MRI anomalies**	Atrophy of CN and hippocampus	No	No	Left lateral ventricular enlargement	Ventricular enlargement, calcific meningioma	Ventricular enlargement, arachnoid cyst	Cerebral cortex and CN atrophy	Atrophy of CN and hippocampus	Atrophy of CN	No	No	Atrophy of CN
**Axonal polyneuropathy**	Sensori-motor	Sensory	Sensory	Sensory	n.a.	Sensory	Sensori-motor	Sensory	No	Sensori-motor	Sensori-motor	Sensori-motor
**EMG pattern**	Chronic denervation with myopathic MUP	Normal	Myopathic MUP	n.a.	n.a.	n.a	Active and chronic denervation	Active and chronic denervation	Normal	Myopathic MUP with chronic denervation	Myopathic MUP with chronic denervation	Myopathic MUP with chronic denervaion
**Muscle weakness (onset age)**	Lower limb-girdle(35)	No	Lower limb-girdle (29)	No	Lower limb-girdle(47)	Lower limb-girdle(37)	Proximal and distal muscles LL (39)	Lower limb-girdle(38)	Lower limb-girdle (53)	Upper and lower limb girdle (40)	Upper and lower limb girdle (45)	Upper and lower limb girdle (40)
**other ** **muscular signs and symptoms**	No	No	n.a.	n.a.	n.a.	n.a.	Fasciculations, cramps, muscle atrophy	Fasciculations, cramps muscle atrophy	No	Cramps at LL	Cramps at LL	Cramps at LL
**Muscle biopsy**	Type-grouping, multi-minicore fibers	n.a.	n.a.	n.a.	n.a.	n.a.	Bizarre fibers, atrophic fascicles	Type-grouping, core fibers	n.a.	Fiber size variability, rimmed vacuoles	Fiber size variability, inflammatory infiltrates	Fiber size variability, increased central nuclei
**Cardiac ** **anomalies (onset age)**	No	No	paroxystic tachycardia (7)	No	paroxystic atrial fibrillation (36)	mild conductive anomalies (39)	No	No	No	YES (57)	No	No

**Table 2:** ChAc patients: molecular findings. *VPS13A* variants on cDNA, their allele frequency in the GnomAD database, variant type and predicted protein change. § Missense variants in *VPS13A* are usually benign and the p.K1198Q variant is anyhow in cis with the pathogenic frameshift c.1114_1115del variant. NF: Variant not found in gnomAD exomes/genomes; ^⁑^ only papers with independent patients quoted; NR: apparently not reported.

Family	cDNA Variant	Exon	Allele Frequency	Variant Type	Protein Change	Previously Reported in ^⁑^
			(gnomAD)			
**A**	c.3817C>T (pat)	34	1 / 251198	Nonsense	p.R1273*	NR
	c.1114_1115del (mat)	13	1 / 250630	Frameshift deletion	p.K372Vfs*4	[8]
	*c.3592A>C (mat)*§	*33*	*3 / 282476*	*Missense*	*p.K1198Q*	*NR* – *likely benign*
**B**	c.3339+4_3339+10delinsTATAGCTGTTATATAAAATTATTTAA	IVS31	NF	Splice-site indel	?	NR
**C**	c.1078C>T	13	3 / 250796	Nonsense	p.Q360*	NR
	c.7867C>T	56	1 / 251190	Nonsense	p.R2623*	[8,12,13,14]
**D**	c.2512+2T>G	IVS24	NF	Splice-site substitution	?	NR
**E**	c.7736_7739del	55	5 / 251030	Frameshift deletion	p.R2579Nfs*26	NR
	c.2825-10T>G	IVS26	NF	Splice-site substitution	?	NR

## Data Availability

All data presented in this study (including primer sequences and detailed experimental methods), if not specified in the Supplementary Material, are available upon request from the corresponding author.

## Supplementary Materials

The following are available online at https://www.mdpi.com/2073-4425/12/3/344/s1, Figure S1: (A) c.1114_1115del; (B) c.3592A>C; (C) c.3817C>T, Figure S2: (A) c.3339+4_3339+10delinsTATAGCTGTTATATAAAATTA TTTAA; (B) exon 30-33 cDNA amplicon, Figure S3: (A) c.1078C>T; (B) c.7867C>T, Figure S4: c.2512+2T>G (homozygous), Figure S5: (A) c.7736_7739del; (B) c.2825-10T>G; (C) effect of the c.2825-10T>G splice-site variant on cDNA sequence, Table S1. Sequencing primers employed for genetic testing of VPS13A., Table S2. Sequencing primers employed for genetic testing of *XK.*

## References

[1] Peikert K., Danek A., Hermann A. (2018). Current state of knowledge in Chorea-Acanthocytosis as core Neuroacanthocytosis syndrome. Eur. J. Med. Genet..

[2] Walker R.H., Jung H.H., Dobson-Stone C., Rampoldi L., Sano A., Tison F., Danek A. (2007). Neurologic phenotypes associated with acanthocytosis. Neurology.

[3] Velayos-Baeza A., Vettori A., Copley R.R., Dobson-Stone C., Monaco A.P. (2004). Analysis of the human VPS13 gene family. Genomics.

[4] Saiki S., Sakai K., Kitagawa Y., Saiki M., Kataoka S., Hirose G. (2003). Mutation in the CHAC gene in a family of autosomal dominant chorea-acanthocytosis. Neurology.

[5] Ishida C., Makifuchi T., Saiki S., Hirose G., Yamada M. (2009). A neuropathological study of autosomal-dominant chorea-acanthocytosis with a mutation of VPS13A. Acta Neuropathol..

[6] Walker R.H., Velayos-Baeza A., Bader B., Danek A., Saiki S. (2012). Mutation in the CHAC gene in a family of autosomal dominant chorea-acanthocytosis. Neurology.

[7] Yi F., Li W., Xie N., Zhou Y., Xu H., Sun Q., Zhou L. (2018). Chorea-Acanthocytosis in a Chinese Family with a Pseudo-Dominant Inheritance Mode. Front. Neurol..

[8] Tomiyasu A., Nakamura M., Ichiba M., Ueno S., Saiki S., Morimoto M., Kobal J., Kageyama Y., Inui T., Wakabayashi K. (2011). Novel pathogenic mutations and copy number variations in the VPS13A gene in patients with chorea-acanthocytosis. Am. J. Med. Genet. B.

[9] Livak K.J., Schmittgen T.D. (2001). Analysis of relative gene expression data using real-time quantitative PCR and the 2(-Delta Delta C(T)) Method. Methods.

[10] Dobson-Stone C., Velayos-Baeza A., Filippone L.A., Westbury S., Storch A., Erdmann T., Wroe S.J., Leenders K.L., Lang A.E., Dotti M.T. (2004). Chorein detection for the diagnosis of chorea-acanthocytosis. Ann. Neurol..

[11] Peluso S., Bilo L., Esposito M., Antenora A., De Rosa A., Pappatà S., De Michele G. (2017). Chorea-acanthocytosis without chorea: Expanding the clinical phenotype. Parkinsonism Relat. Disord..

[12] Dobson-Stone C., Danek A., Rampoldi L., Hardie R.J., Chalmers R.M., Wood N.W., Bohlega S., Dotti M.T., Federico A., Shizuka M. (2002). Mutational spectrum of the CHAC gene in patients with chorea-acanthocytosis. Eur. J. Hum. Genet..

[13] Velayos-Baeza A., Holinski-Feder E., Neitzel B., Bader B., Critchley E.M., Monaco A.P., Danek A., Walker R.H. (2011). Chorea-acanthocytosis genotype in the original Critchley kentucky neuroacanthocytosis kindred. Arch. Neurol..

[14] Niemelä V., Salih A., Solea D., Lindvall B., Weinberg J., Miltenberger G., Granberg T., Tzovla A., Nordin L., Danfors T. (2020). Phenotypic variability in chorea-acanthocytosis associated with novel VPS13A mutations. Neurol. Genet..

[15] Jung H.H., Danek A., Walker R.H. (2011). Neuroacanthocytosis syndromes. Orphanet J. Rare Dis..

[16] Weaver J., Sarva H., Barone D., Bobker S., Bushara K., Hiller A., Ishii M., Jankovic J., Lakhani S., Niotis K. (2019). McLeod syndrome: Five new pedigrees with novel mutations. Parkinsonism Relat. Disord..

[17] Dulski J., Sołtan W., Schinwelski M., Rudzińska M., Wójcik-Pędziwiatr M., Wictor L., Schön F., Puschmann A., Klempíř J., Tilley L. (2016). Clinical variability of neuroacanthocytosis syndromes-a series of six patients with long follow-up. Clin. Neurol. Neurosurg..

[18] Zhu H., Feng X.M., Zhao T., Liu J.Y. (2019). Neuroacanthocytosis with unusual clinical features: A case report. Medicine (Baltimore).

[19] Limos L.C., Ohnishi A., Sakai T., Fujii N., Goto I., Kuroiwa Y. (1982). “Myopathic” changes in chorea-acanthocytosis. Clinical and histopathological studies. J. Neurol. Sci..

[20] Dotti M.T., Malandrini A., Federico A., Danek A. (2004). Neuromuscular findings in eight Italian families with neuroacanthocytosis. Neuroacanthocytosis Syndromes.

[21] Dubowitz V., Sewry C., Oldfors A. (2013). Muscle Biopsy: A Practical Approach.

[22] Melone M.A., Di Fede G., Peluso G., Lus G., Di Iorio G., Sampaolo S., Capasso A., Gentile V., Cotrufo R. (2002). Abnormal accumulation of tTGase products in muscle and erythrocytes of chorea-acanthocytosis patients. J. Neuropathol. Exp. Neurol..

[23] Melone M.A.B., Peluso G., Danek A. (2004). Substrates for Transglutaminase-Catalyzed Cross-Linking: Relevance to Pathogenesis of Huntington’s Disease and Chorea-Acanthocytosis. Neuroacanthocytosis Syndromes.

[24] Saiki S., Sakai K., Murata K.Y., Saiki M., Nakanishi M., Kitagawa Y., Kaito M., Gondo Y., Kumamoto T., Matsui M. (2007). Primary skeletal muscle involvement in chorea-acanthocytosis. Mov. Disord..

[25] Danek A., Jung H.H., Melone M.A., Rampoldi L., Broccoli V., Walker R.H. (2005). Neuroacanthocytosis: New developments in a neglected group of dementing disorders. J. Neurol. Sci..

[26] Swash M., Schwartz M.S., Carter N.D., Heath R., Leak M., Rogers K.L. (1983). Benign X-linked myopathy with acanthocytes (McLeod syndrome). Its relationship to X-linked muscular dystrophy. Brain.

[27] Hewer E., Danek A., Schoser B.G., Miranda M., Reichard R., Castiglioni C., Oechsner M., Goebel H.H., Heppner F.L., Jung H.H. (2007). McLeod myopathy revisited: More neurogenic and less benign. Brain.

[28] Kawakami T., Takiyama Y., Sakoe K., Ogawa T., Yoshioka T., Nishizawa M., Reid M.E., Kobayashi O., Nonaka I., Nakano I. (1999). A case of McLeod syndrome with unusually severe myopathy. J. Neurol. Sci..

[29] Danek A., Witt T.N., Stockmann H.B., Weiss B.J., Schotland D.L., Fischbeck K.H. (1990). Normal dystrophin in McLeod myopathy. Ann. Neurol..

[30] Carter N.D., Morgan J.E., Monaco A.P., Schwartz M.S., Jeffery S. (1990). Dystrophin expression and genotypic analysis of two cases of benign X linked myopathy (McLeod’s syndrome). J. Med. Genet..

[31] Jung H.H., Russo D., Redman C., Brandner S. (2001). Kell and XK immunohistochemistry in McLeod myopathy. Muscle Nerve.

[32] Roulis E., Hyland C., Flower R., Gassner C., Jung H.H., Frey B.M. (2018). Molecular Basis and Clinical Overview of McLeod Syndrome Compared With Other Neuroacanthocytosis Syndromes: A Review. JAMA Neurol..

[33] Danek A., Rubio J.P., Rampoldi L., Ho M., Dobson-Stone C., Tison F., Symmans W.A., Oechsner M., Kalckreuth W., Watt J.M. (2001). McLeod neuroacanthocytosis: Genotype and phenotype. Ann. Neurol..

[34] Nishida Y., Nakamura M., Urata Y., Kasamo K., Hiwatashi H., Yokoyama I., Mizobuchi M., Sakurai K., Osaki Y., Morita Y. (2019). Novel pathogenic VPS13A gene mutations in Japanese patients with chorea-acanthocytosis. Neurol. Genet..

[35] Critchley E.M., Clark D.B., Wikler A. (1968). Acanthocytosis and neurological disorder without betalipoproteinemia. Arch. Neurol..

[36] Karczewski K.J., Francioli L.C., Tiao G., Cummings B.B., Alföldi J., Wang Q., Collins R.L., Laricchia K.M., Ganna A., Birnbaum D.P. (2020). The mutational constraint spectrum quantified from variation in 141,456 humans. Nature.

