# Supplementary material for: Neuroacanthocytosis Syndromes in an Italian Cohort: Clinical Spectrum, High Genetic Variability and Muscle Involvement
Source: Genes (Basel). 2021 Feb 26;12(3):344. doi: 10.3390/genes12030344 (PMC7996727; doi:10.3390/genes12030344)
Supplement: Supplementary file 1 [file genes-12-00344-s001.pdf]

SUPPLEMENTARY MATERIAL

Detailed clinical history and laboratory results

Family A

The proband (A-II:4) first came to medical attention at the age of 6 for the subtle onset of attention deficit and behavioral dysfunctions with irritability; during adolescence, he developed subtle involuntary movements of the four limbs with frequent guttural vocalizations and, when he was 23 years old, he presented with tonic-clonic seizures. Two years later a neurological examination revealed a hyperkinetic syndrome with choreoathetosic movements of the four limbs and impersistence of tongue protrusion, generalized hypotonia and hyporeflexia. Serum CK at rest was >600 UI/L. At the age of 37 he underwent an extensive clinical and diagnostic re-evaluation: at that time, the neuropsychological examination showed a deficient global cognitive performance with a total Mini Mental State Examination (MMSE) score of 22.75, deficits in visuo-spatial skills and executive functions (working memory and planning). Neurophysiological tests showed a sensori-motor axonal polyneuropathy with chronic denervation and rare myopathic motor unit potentials (MUP).Electroencephalogram (EEG) showed epileptiform abnormalities on a background of globally slowed brain activity. Brain MRI showed bilateral atrophy of the caudate nucleus and hippocampus, with hemosiderin deposits in the basal and red nuclei. Peripheral blood smear showed 19.2% of acanthocytes and chorein was not detectable by Western blotting.

The younger brother (A-II:5) was evaluated at the age of 25, after ChAc diagnosis was established in A-II:4. His medical history was irrelevant but one year earlier he had developed an anxiety disorder with panic attacks and claustrophobia, for which he is currently treated with psychotherapy. Neurological examination revealed a hyperkinetic syndrome with akathisia, impersistence of tongue protrusion and sporadic tics such as "throat cleaning" and "sniffing"; hyporeflexia was also present. Serum CK levels reached 1971 UI/L, despite no complaints of muscular symptoms and normal muscle strength. Acanthocytes were found in 8.3% of cells on peripheral blood smear. Neurophysiological tests revealed the presence of sensory axonal polyneuropathy along with a diffuse active denervation. Brain MRI and electroencephalogram were unremarkable.

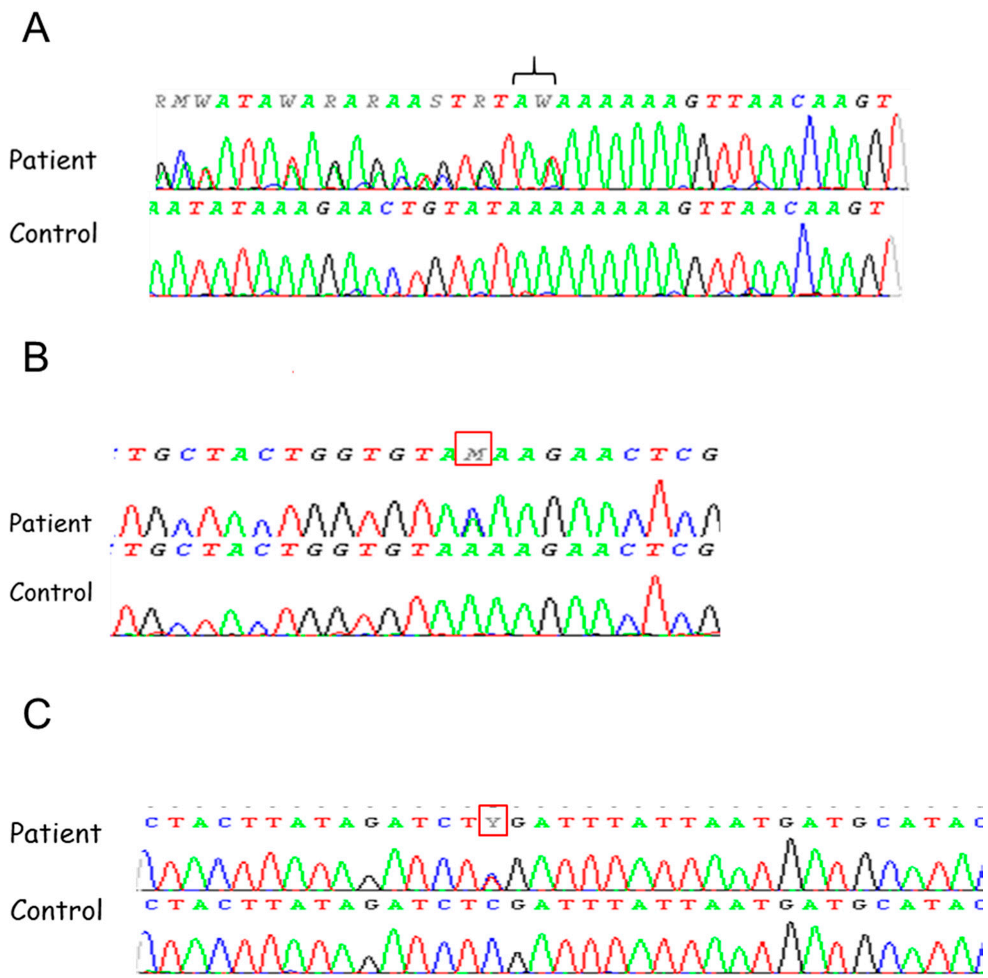

Supplementary Figure 1: A) c.1114_1115del; **B)** c.3592A>C; **C)** c.3817C>T

Family B

The proband (B-II:3) suffered paroxysmal tachycardia at the age of 7 and, for this reason, he was treated with surgical ablation at the age of 14. Neurological symptoms started at 27 years with orofacial tics. In the following years he developed generalized choreic movements and cervical tics, which caused traumatic cervical myelopathy when he was 29 years old. Brain MRI showed no relevant findings, while serum CK was increased (489 UI/L). Frequent hospitalizations followed, with consistently high CK (up to 948 UI/L) and evidence of myopathy and sensory polyneuropathy by electromyography. At the age of 30, acanthocytes (8.6%) were eventually observed in the blood smear.

The other affected brother (B-II:5) initially showed orofacial tics at the age of 25. A few months later he also suffered generalized tonic-clonic seizures. Mood disorder, anxiety and insomnia started shortly thereafter. After ChAc was diagnosed in his older brother, B-II:5 was thoroughly evaluated at the age of 26: clinical examination revealed bilateral impaired finger tapping and decreased deep tendon reflexes. Electromyography and laboratory analyses were also performed, revealing sensory axonal polyneuropathy with increased serum CK (5564 UI/L) and acanthocytes in the blood smear (3.1%).

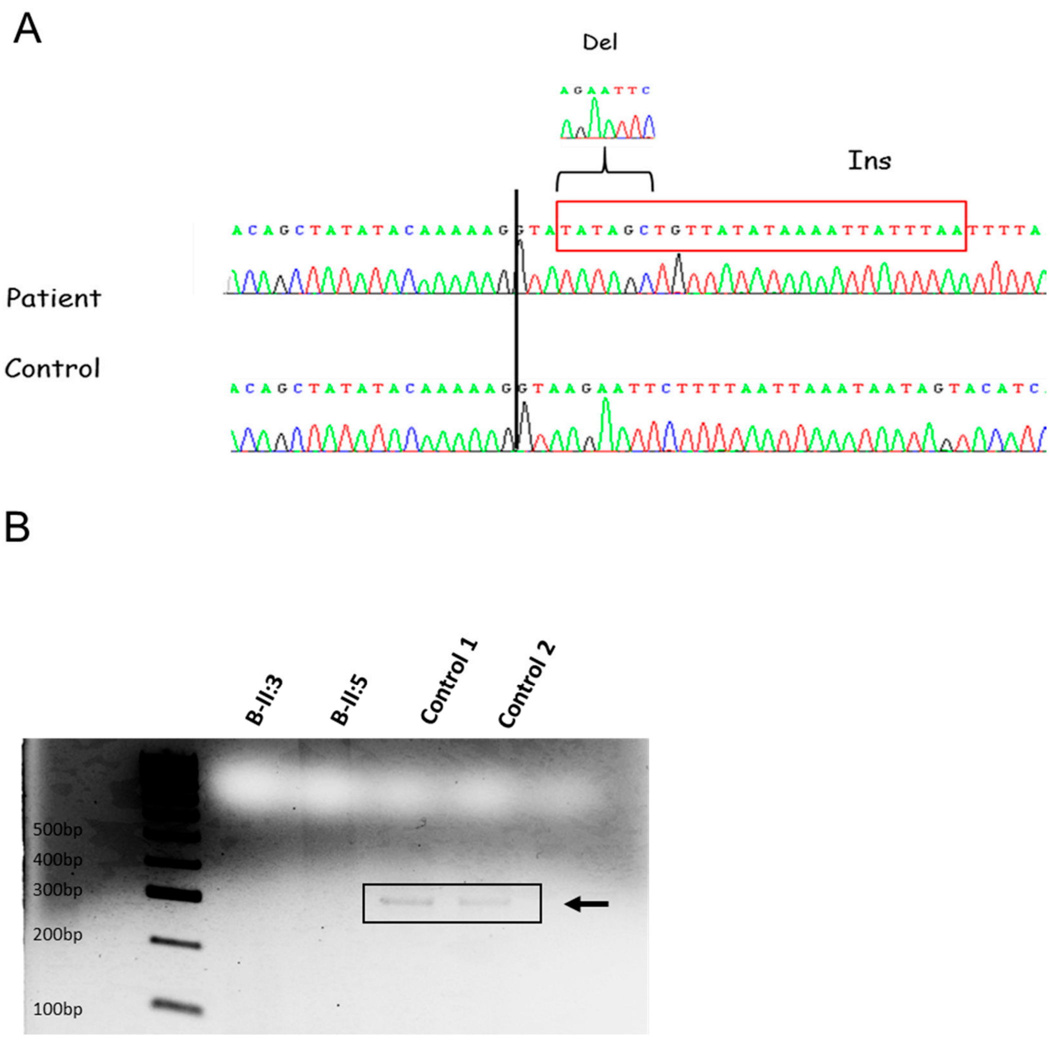

Supplementary Figure 2: A) c.3339+4_3339+10delinsTATAGCTGTTATATAAAATTA TTTAA; **B) e**xon 30-33 cDNA amplicon.

Family C

The younger brother (C-II:4) had an earlier neuropsychiatric onset at the age of 20, with psychosis and obsessive-compulsive disorder, apparently after a primary herpetic gingivostomatitis followed by viral encephalitis. Shortly afterwards, mood disorder, motor tics, involuntary movements of the upper limbs and trunk progressively appeared. Over the years, the condition worsened with the onset of parkinsonian symptoms, such as freezing, postural instability and bradykinesia. At 34 years of age, neurophysiologic evaluation revealed a sensory axonal polyneuropathy and acanthocytes were detected. Molecular tests for NA were eventually performed and, while MLS was excluded, western blot analysis showed a marked reduction of chorein, establishing the ChAc diagnosis. In the following years, due to severe dysphagia for solids and liquids, percutaneous endoscopic gastrostomy was performed.

The older brother (C-II:3) had some motor tics when he was a child and a single episode of seizures at 11 years of age. When he was 30, he started displaying choreo-dystonic movements of the limbs, mouth and tongue, facial dyskinesias and grimace type automatisms, later associated with phonation disorder, dysphagia, drooling, bruxism, loss of deep tendon reflexes and gait disturbance. At the age of 35 seizures reappeared and were treated with phenobarbital. During hospitalization for seizures with loss of consciousness, paroxysmal atrial fibrillation was noticed and treated with amiodarone. At the age of 40 his nutritional problems worsened with loss of weight due to both hyperkinesia and appetite reduction. Over the years, serum CK levels were constantly increased (up to 3435 UI/L).

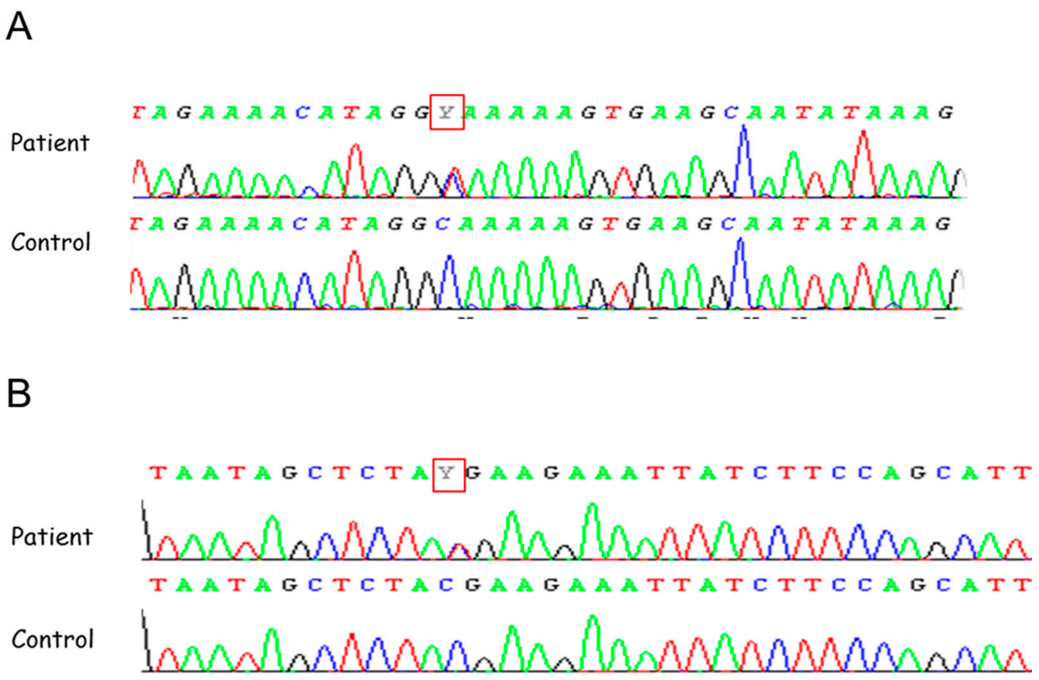

Supplementary Figure 3: A) c.1078C>T; **B)** c.7867C>T

Family D

Proband D-II:2 first came to medical attention at the age of 40, for the subtle onset of generalized weakness (MRC Scale: 4/5), with muscle cramps and diffuse fasciculations. Under resting conditions, serum CK was >3000 UI/L; a neurophysiological evaluation showed axonal sensori-motor polyneuropathy with active multifocal denervation. In the subsequent decade, muscle weakness progressed to a greater deficit of strength in the upper limbs (MRC scale: 2/5) and lower limb paresis, in the context of severe muscular atrophy. He became dysphagic and hypo-dysphonic; there were buccolingual choreic movements, with frequent phonic tics (including simple, short vocalizations). For the worsening dysphagia, percutaneous endoscopic gastrostomy was performed at the age of 52 and he underwent an extensive clinical re-evaluation. His cognitive function was globally compromised (MMSE 24/30, Frontal Assessment Battery 10/18). Brain MRI showed atrophy of the cerebral cortex, basal gangliain the T1-weighted sequences with hyperintensity of the basal gangliain the T2- and FLAIR-weighted sequences and hypointensity in the T2*w sequences. The peripheral blood smear showed 17% acanthocytes; chorein was not detectable by Western blotting. He eventually died at the age of 57 for pneumonia, leaving two healthy (presumed heterozygous) sons. The younger brother (D-II:3) also displayed NA symptoms at the time of family screening. He had been previously reported by Peluso et al. [10] and, according to clinical records, suffered a sudden onset of generalized and focal epileptic seizures at the age of 30; afterwards he developed muscle weakness with widespread fasciculation, cramps and elevated serum CK (1522 UI/L). He also suffered from obstructive sleep apnea. At the age of 46 he was able to stand up and walk without assistance, even if with some instability; he presented with diffuse fasciculations and was areflexic. A neurophysiological evaluation showed sensory axonal neuropathy and active multifocal muscle denervation and fasciculation. Brain MRI showed basal nuclei atrophy, without signal alteration; the left hippocampal cortex was reduced in volume and showed T2 hyperintensity, compared to the right one, which was attributed to mesial temporal sclerosis. Electroencephalography displayed slow and isolated epileptic abnormalities in frontal left-temporal areas. His cognitive status showed mild cognitive impairment with deficits in memory, attention and language. Acanthocytes on peripheral blood smear were 13%. At the age of 48, II:3 had episodes of feeding dystonia, dysphagia, drooling, generalized movement slowness and worsening of language and cognitive functions. Seizures did not benefit from lacosamide and levetiracetam treatment. The neurological examination showed waddling gait, mild hypomimia, orolingual tremor, upper limb postural tremor and dystonia, rigidity and bradykinesia prevalent at the left limbs, decreased vibration sense and absent deep tendon reflexes. Parkinsonism was poorly responsive to L-DOPA whereas sublingual atropine improved drooling. The last neuropsychological assessment showed severe impairment of memory, visuospatial and executive functions, as well as ideomotor and constructive praxis.

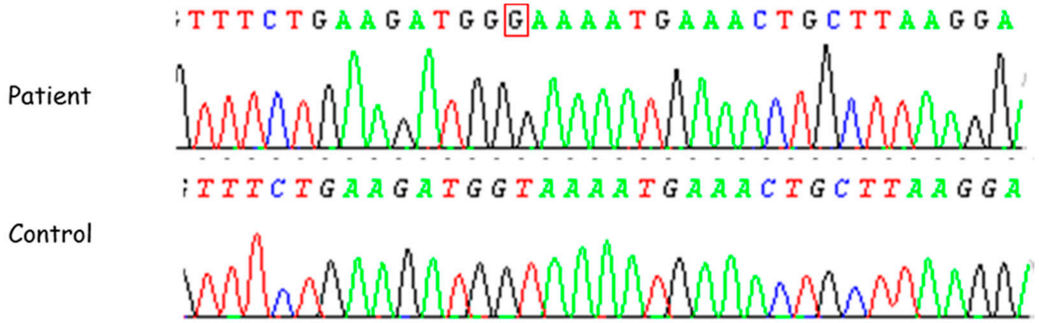

Supplementary Figure 4: c.2512+2T>G (homozygous)

Family E

Acanthocytes (>40%) had been found in three blood smears of the proband (E-II:2), who is the only patient of his family. At the age of 28, he developed severe depression and personal carelessness. His psychiatric picture progressively worsened and he became socially withdrawn with apathy, anhedonia, and pathological gambling at the age of 40 years. His phenotype then evolved and he presented with hypophonia, drooling and generalized motor slowing. A neurological evaluation showed waddling gait, hypomimia, slight hand and tongue tremor, bilateral bradykinesia and areflexia. Serum CK levels were consistently elevated (>1500 UI/L). A nerve-conduction study, electromyography and EEG showed no abnormalities, but brain MRI showed mild atrophy of the caudate nuclei. Furthermore, a DaT-scan showed a diffuse and symmetric decrease of the striatal radiotracer uptake [10]. Neuropsychological evaluation documented only a deficit in executive functions and his Mini-Mental Status Exam score was 28/30. A six-month treatment with L-DOPA did not improve his motor symptoms. Afterwards, he developed a progressive lingual, mandibular and pharyngeal dystonia that required percutaneous endoscopic gastrostomy for nutrition and severely reduced his ability to speak.

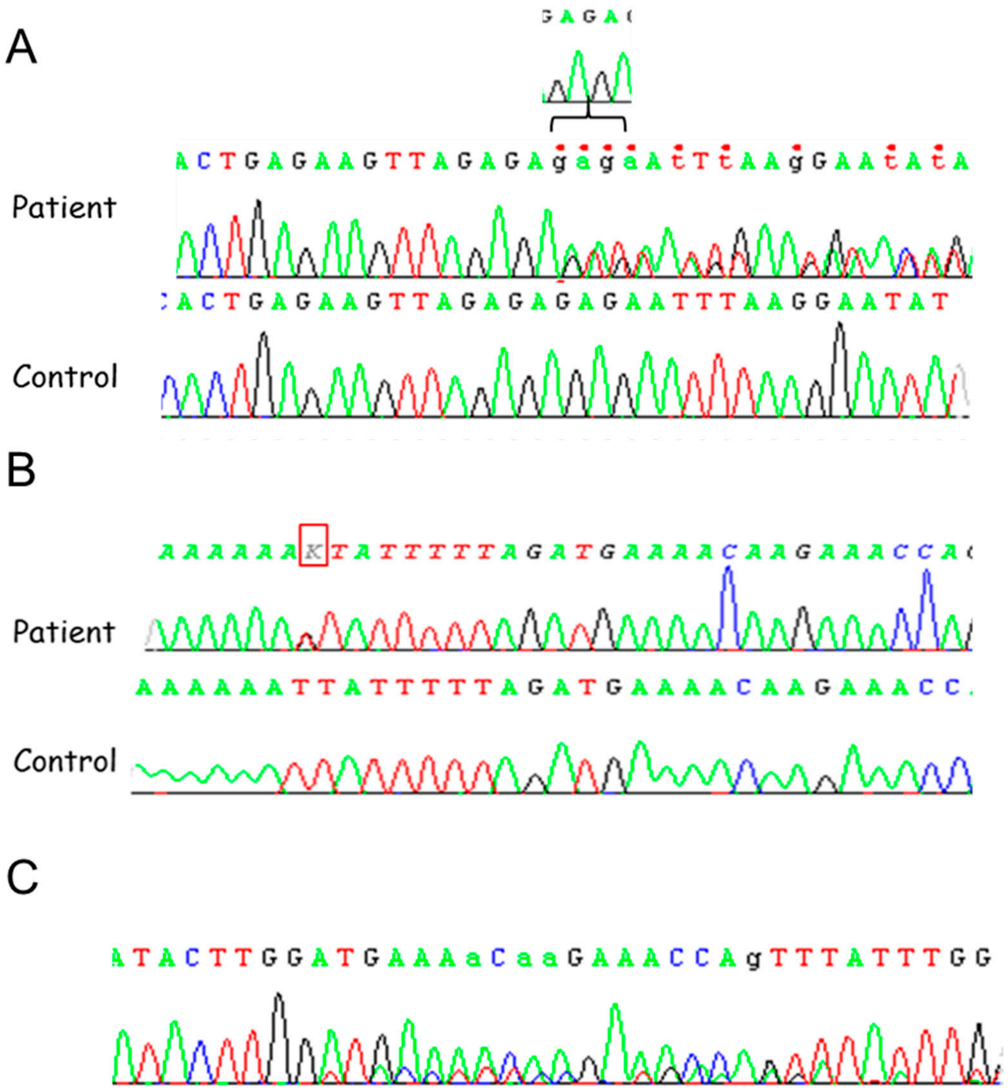
A
GAGA
ACTGAGAAGTTAGAGAgagaAtTtAAgGAAtatA
Patient
ACTGAGAAGTTAGAGAGAGAATTTAAGGAATAT
Control
B
AAAAAAKTATTTTTAGATGAAAACAAGAAACCA
Patient
AAAAAATTATTTTTAGATGAAAACAAGAAACC
Control
C
ATACTTGGATGAAAaCaaGAAACCAgTTTATTTGG

Supplementary Figure 5: A) c.7736_7739del; **B)** c.2825-10T>G; **C)** effect of the c.2825-10T>G splice-site variant on cDNA sequence.

Family F

Proband III:5 came to our observation at the age of 63, four years after the onset of choreic movements of the trunk and limbs. He had generalized seizures at 39 years of age that were treated with phenobarbital and his serum CK levels were repeatedly increased (>1000 UI/L). Neurological examination showed choreic movements of the face, trunk and limbs, with secondary alteration of gait; there was also a moderate strength deficit (MRC: 4/5) with proximal muscle hypotrophy, diffuse hypotonia and areflexia. Neurophysiological tests showed sensory-motor axonal polyneuropathy; electroencephalogram showed diffuse epileptic abnormalities. His red blood cells had a kk type, Kx antigen was undetectable and 18% of them were acanthocytes.

Information was collected from available clinical records of F-III:4 and F-III:9. Patient III:4 came to medical attention in his fifth decade with muscle fatigability and a long history of hyperCKemia (2500 UI/L). Neurological examination showed mild muscle weakness with hypotrophy of scapular and pelvic girdles and diffuse areflexia. Neurophysiological examination revealed sensory axonal polyneuropathy and diffuse muscle denervation. Electroencephalogram and brain MRI were unremarkable, while 10% acanthocytes in peripheral blood were found. In 2007, following a cardiac arrest, III:4 received a diagnosis of dilatated cardiomyopathy and a pacemaker was implanted. He deceased at 60 years-old due to heart failure. The clinical history of patient III:9 started at the age of 20 with a behavioral disorder characterized by anxiety, restlessness and alcohol and drug abuse. Afterwards he developed involuntary movements of the trunk and the limbs, along with asymptomatic hyperCKemia (approx. 1000 UI/L). At the age of 40, III:9 was reassessed after the onset of generalized convulsive seizures and neurophysiological studies revealed diffuse neurogenic signs and reorganization of motor units along with small MUPs. Peripheral blood smear showed 7% acanthocytes. After the age of 45, the patient gradually developed diffuse muscle weakness and hypotrophy with gait impairment, worsening of choreic movements, dysarthria, dysphagia and trunk and oro-facial dystonia. Serum CK levels were further elevated (1200-8000 UI/L) and brain MRI revealed bilateral caudate nuclei atrophy. III:9 required percutaneous endoscopic gastrostomy due to severe dysphagia at the age of 47 and died at the age of 50 for ab ingestis pneumonia.

Table S1. Sequencing primers employed for genetic testing of *VPS13A*.

***VPS13A* primer**	**Primer sequence (5′ to 3′)**	**length**
VPS13A – 1Fn	TCGGAAAATATAGACCTTTTC	21
VPS13A – 1Rn	GCAGACTTGCGCACGCGT	18
VPS13A – 2F	TAGGCAGATTATATTATCTGTTATG	25
VPS13A – 2R	TCAGGGAGATTAAAACTCCATTTA	24
VPS13A – 3F	AAGTAAAGAG-<br>TATTCATTTATGTTGT	26

VPS13A – 3R	CACATAATAGATA-TATTAGGCAAATT	26
VPS13A – 4F	AAATAGACTAACCA-TAAATGCAGG	24
VPS13A – 4R	AAAGTATTTTGAATATAAA-TATTTTTCC	28
VPS13A – 5Fn	CCTCATGATTCGCCCAGCT	19
VPS13A – 5Rn	ACAAACCACATTAAAATTCCCC	22
VPS13A – 6F	CAGCAAGGCAACGTAAGCATG	21
VPS13A – 6R	TGGGATTTAACAGATTGGCCATT	23
VPS13A – 7F	CCAGAGTGCTGGGATTACAGA	21
VPS13A – 7R	CACCTATTTCTGATGCTGGCAT	22
VPS13A – 8F	CATGAAAGGGACATTGGTCTAG	22
VPS13A – 8R	AAAATTCACATA-CATCCTTATCTATA	26
VPS13A – 9F	ACTCTGTGATATGGCTCACTTAA	23
VPS13A – 9R	GCCTAGGAAAAAATACTGACTAC	23
VPS13A – 10Fn	GCAGTGAGCTGAGATTGCAC	20
VPS13A – 10Rn	TTTTGTTGGACATTTTCCCC	20
VPS13A – 11F	GGAAATCAGTGTTTTCAACTTCAT	24
VPS13A – 11R	GAAACATTTAAAATCTTAGTTTT-GCT	26
VPS13A – 12F	CCTAAAAAGTCAG-TAATGTAACTTT	25
VPS13A – 12R	GTTTGTATCAGACCTTAAAAATGG	24
VPS13A – 13F	CCTAATCTTT-GTATTATAGTTATGTA	26
VPS13A – 13R	AATGAAAGGTAGGGAGTATCAGT	23
VPS13A – 14F	TAGTGCAGCCATTTGATAGATTAT	24
VPS13A – 14R	AACCACTTTTTAAACAATATGTT	23
VPS13A – 15Fbis	TGCAGTTTCTGTGTTCTGTTATTA	24
VPS13A – 15Rbis	ACTAAATCTCTGGGCATAAAGCT	23

VPS13A – 16F	CAA-GAAAGTAAATTTCACATTCTGT	25
VPS13A – 16-17R	GGATATATGAAACTATAGTGCTCT	24
VPS13A – 17F	GGTGGTTTTTGAAATGAATGTACT	24
VPS13A – 17R	GGATATATGAAACTATAGTGCTCT	24
VPS13A – 18F	AGAAATGCAAAGTGAAGTTGTCTT	24
VPS13A – 18Rb	CTGTTAAAA-TAAACATCATTAACACA	26
VPS13A – 19Fb	TATCATAGAAGCTGAACCTTTAGA	24
VPS13A – 19R	TAAACTTTACTAAAAACATTC-TACCA	26
VPS13A – 20FBis	CAGTTCATATATTTAGTGATTCTGT	25
VPS13A – 20RBis	CACCCCAAATGCTGTATCATAAA	23
VPS13A – 21F	AAGTCTAAAAATTAAACAC-TTTATACT	27
VPS13A – 21R	ACGTTCTAACACATTTAAGAGGAA	24
VPS13A – 22F	TTCCTCATATAATGGAATGTGTGA	24
VPS13A – 22R	GTTAAGTATGGTATTTCCCTAGC	23
VPS13A – 23F	GTTTAAAATCACATTTGAT-TCAGATA	27
VPS13A – 23R	CAAATTGCAAATTGTCTTATA-TAAAAT	27
VPS13A – 24F	AACTGTTGTAGAACTTTGAA-TAAAC	25
VPS13A – 24R	AAAAGGAAAAGATATTTTCCTTCC	24
VPS13A – 25F	AAGAGAGCCTTAGTGTTTTAGTG	23
VPS13A – 25R	TAGGGTGAAAAAGATAGTGCTTC	22
VPS13A – 26R	AAATGTACTGATTCAGAGCCTAAA	24
VPS13A – 27F	GCCTTCTTTTGCAATTACATAATTA	25
VPS13A – 27R	CAAAGAGATTATGTTACAG-TGAAG	24
VPS13A – 28Fn	GGACTATAATGTGATTGTATATG	23
VPS13A – 28Rn	CACTATGCTTTGTGGCATAAAG	22

VPS13A – 29F	ATATCCTTTATGCCACAAAGCATA	24
VPS13A – 29R	CTGAAGCCAATATTT-GAAGTTAATA	25
VPS13A – 30F	ACTATTTGTGGTGATATTTCAGTTA	25
VPS13A – 30Rb	TAGCTTTGCGTTTATTTCAGTTTC	24
VPS13A – 31Fb	GATCAGAAATGTAACATTTCTGAA A	25
VPS13A – 31Rbis	ATGCACATAATAAGCCATACTACT	24
VPS13A – 32F	TGGCTTGTGAATACTTGGGAGA	22
VPS13A – 32R	GAATTCTTACTAGTCTTCATCCAT	24
VPS13A – 33F	GGTAAAACCATAAA-TATCAATATATAT	27
VPS13A – 33R	GTAAACAGACTACTCAAGACATC	23
VPS13A – 34F	ACCCAACAAATTATTGATTTCTG	23
VPS13A – 34R	TCCACTCAAATGATGCCTTATGT	23
VPS13A – 35F	GAATTTTAACTGCAG-TTAAATTCTG	25
VPS13A – 35R	GATTAGAAACAGAT-TTACAAATCTTA	26
VPS13A-36Fnew	AAGAGTCCTAAGCCAGAAGTTAC	23
VPS13A-36Rnew	ATAACTCATTTAAAAGGGAAA-TAGTTA	27
VPS13A – 37F	GCAATAATTTGTAGTAGTTCAT-ATC	25
VPS13A – 37R	GCAATTCTGGAGATGAATTCTGT	23
VPS13A – 38F	GAGATAAGAGGTCTTTGAGTTTTA	24
VPS13A – 38R	CTAAAAGGCTAATCAAGGTTAAGT	24
VPS13A – 39F	GTAGTTGATTCCTTTGGGATCTA	23
VPS13A – 39R	TAGGTAGTTTTTGTTTCCTAAAATG	25
VPS13A – 40F	CATACCTATTACTAGGAAAGTCTT	24
VPS13A – 40R	AATTAAAAGACTTGCTATAA-TAACTTT	27

VPS13A – 41F	GGTAGTAATGTTAATTCAG-TATTTAA	26
VPS13A – 41R	CACAAGATTCCATAA-TATTCCATAT	25
VPS13A – 42F	ACTGAATGGCATGTAGACTGGT	22
VPS13A – 42R	GCTGTGAACCTAAATCTGTTCTT	23
VPS13A – 43F	CTTTCTAATGTTGGTATTGGGATT	24
VPS13A – 43R	TGACAACTAAGTTATTAAACTTCT TA	26
VPS13A – 44F	CTGTTCTCCTTGTCATTTAATTTTA	25
VPS13A – 44R	CTTGCTAAGATTCTACAAAAGAGT	24
VPS13A – 45F	AGTTTAA-GAATCATCTGAAATTTCTA	26
VPS13A – 45R	TATATTCTTTCAGTAATAATTT-GTTGT	27
VPS13A – 46F	TGATAGTTCCTTTGTTAAGATAGTT	25
VPS13A – 46R	CTATTAGCAGGATGGATTTGATC	23
VPS13A – 47F	GTTATTCTAAAGCTGTAATTATA-TAG	26
VPS13A – 47R	TAAAA-TAGTTTTTATTGATCTTAATCTT	28
VPS13A – 48F	GAATACATAAATAGAATTTTGAGG	25
VPS13A – 48R	AACAAGTTATTTCTATCCAAGTTTC	24
VPS13A – 49F	TTTATGCTAAAAAGTAATTTATCA GTT	27
VPS13A – 50R	GAGTTAAAACATTACATGGTGACT	24
VPS13A – 51F	TCTATTCTGATGGGAATATTAA-GAT	26
VPS13A – 51R	AGGG-GAAAAATCAACAGAAGTAAT	24
VPS13A – 52F	TTCTCAG-TCATCCCAAAAATTAAAT	24
VPS13A – 52R	CACTGTTTTACACAGCTACAATG	23

VPS13A – 53Fn	TCAGAACGATCACAGATCTCAGT	23
VPS13A – 53Rn	CCAGGCTGATCTCCAACTCA	20
VPS13A – 54F	AATCTAAATTTCATGTTCTTTGGG	23
VPS13A – 54R	TCATTTGAGATTTTACAA-TAACAATTT	27
VPS13A – 55F	GTTCTGAAATTTTAGTGAAGGTAT	24
VPS13A – 55R	CTTTCCTTAAATTTCACTAGTGATT	25
VPS13A – 56F	ACATGGGATATTAA-GATCTAAATCT	25
VPS13A – 56R	AGCACAACATTGAAACTGAA-TACT	25
VPS13A – 57F	TGAGCCACCGTGCTTGGTCA	20
VPS13A – 57Rbis	TGCTGGTCCTTTTTCAAGCAGT	22
VPS13A – 58F	TGAAATTAAGATTGTAGTAGAT-TTTG	26
VPS13A – 58R	ATCTTTGACTTGTCCAACATTTAAA	25
VPS13A – 59F	TTAAAATTTAGTCAACTAAA-TAGTCC	26
VPS13A – 59R	TGCAACAAAGTCATTTTAATAC-CTTT	26
VPS13A – 60F	GTTGAGTCTGGATCTTATAGAAG	23
VPS13A – 60R	ACAGATTTTATATATTACTGAA-GAACA	27
VPS13A – 61F	AACTTT-GAAATCTTATTTATGGTGTA	26
VPS13A – 61R	TGATCTGCAACGTACTTCACAC	22
VPS13A – 62F	ACTAGAAGGAAAGGTTTGGAGAA	23
VPS13A – 62R	TGGCTATTTAGTTCCTCAATAGTT	24
VPS13A-63Fnew	AGGCAGAGGTTGCAGTGAGC	20
VPS13A-63Rnew	CCTCTTACCATTGTTTGCTGCA	22
VPS13A – 64F	TGATATTACTTCCTCTGGGACAA	23
VPS13A – 65R	GTGAGAATCATGTCTATTTCCTTA	24
VPS13A – 66F	GTGTAATCCAAACTTGGTTCTTC	23

VPS13A – 67R	GCCTGAAGCCAAAGAGCAGAT	21
VPS13A – 68F	TAATCTGAAATAA-GATTGTTTTTAGTA	27
VPS13A – 68R	CAAA-TAATTTAATATATATTAAATGGCT	28
VPS13A – 69F	CTCTATGTTAATGGTTTGCCCC	22
VPS13A – 69R	ACTATATGGAACAAAAGGTGAA-TAA	25
VPS13A – 70F	TGCATTTGCACTTGCGATTCATT	23
VPS13A – 70R	GACATCTATAAAAGAGGTAGAGC	23
VPS13A – 71F	GTATAAGAGGGACACTTTAGGC	22
VPS13A – 71R	TTACATATACTTTTAAAAC-CTGCCA	25
VPS13A – 72F	TTGGCTGTCAGTATTACACCTAA	23
VPS13A – 72R	TCATTTCTGTAATATGCTTCCCAA	24

Table S2. Sequencing primers employed for genetic testing of *XK*.

***XK* primer**	**Primer sequence (5′ to 3′)**	**length**
XK-1F	GCATGCTGGGAGCCCCTCG	19
XK-1R	CTCAGCCACCTGTCTCCACA	20
XK-2F	GTCAAGGCTTTAAGAATCAGGC	22
XK-2R	AAGTGCACTTAAGAACAAATTTCC	24
XK-3F	GCTGCAGTAGGATTCCTAGTG	21
XK-3R	CCTGTGTAGGGCTCATTATTTG	22
